# Nine new species of the spider genus *Stedocys* (Araneae, Scytodidae) from China and Thailand

**DOI:** 10.24272/j.issn.2095-8137.2017.066

**Published:** 2017-09-18

**Authors:** Jiang-Lang Wu, Yu-Fa Luo, Shu-Qiang Li

**Affiliations:** ^1^Southeast Asia Biodiversity Research Institute, Chinese Academy of Sciences, Yezin Nay Pyi Taw 05282, Myanmar; ^2^Institute of Zoology, Chinese Academy of Sciences, Beijing 100101, China; ^3^School of Life and Environment Sciences, Gannan Normal University, Ganzhou Jiangxi 341000, China; ^4^University of Chinese Academy of Sciences, Beijing 100049, China

**Keywords:** Taxonomy, Morphology, Diagnosis, Biodiversity, Caves

## Abstract

Nine new species of the genus *Stedocys* Ono, 1995 are described: *Stedocys gaolingensis* Wu & Li sp. n. (♂♀, Guangxi), *S. huangniuensis* Wu & Li sp. n. (♀, Guangxi), *S. ludiyanensis* Wu & Li sp. n. (♂♀, Guangxi), *S. matuoensis* Wu & Li sp. n. (♀, Guangxi), *S. pulianensis* Wu & Li sp. n. (♂, Guangxi), *S. shilinensis* Wu & Li sp. n. (♂♀, Hainan), *S. xianrenensis* Wu & Li sp. n. (♂♀, Guangxi), *S. xiangzhouensis* Wu & Li sp. n. (♂♀, Guangxi) from China, and *S. zhaoi* Wu & Li sp. nov. (♂♀, Kanchanaburi) from Thailand. Diagnoses of nine new species are provided. DNA barcodes for six new species are documented for future use and as proof of molecular differences between these species.

## INTRODUCTION

The genus *Stedocys* was established by Ono (1995) based on male of *S. uenorum* Ono, 1995 collected from a cave in Thailand. Thus far, this genus comprises only three species: *S. uenorum* from Thailand, *S. leopoldi* (Giltay, 1935) from Malaysia and Thailand, and *S. pagodas* Labarque et al., 2009 from China (World Spider Catalog, 2017). According to Ono (1995), the genus *Stedocys* is diagnosed by a short tarsus and relatively long bulb with an aciculate embolus. Diagnosis of the genus *Stedocys* may change based on molecular phylogenetic study if fresh material of *S. uenorum* available. However, it is not possible to recollect *S. uenorum* until now because no detailed information on the type locality.

In this paper, we describe nine new species of the genus collected in caves from China and Thailand. Morphological descriptions and photos are given for all new species.

## MATERIAL AND METHODS

Specimens were examined and measured with a LEICA M205 C stereomicroscope. Images were captured with an Olympus C7070 wide zoom digital camera (7.1 megapixels) mounted on an Olympus SZX12 dissecting microscope, and they were montaged using Helicon Focus 6.6.1 image stacking software (Khmelik et al., 2006). Male and female genitalia were examined and photographed after dissection. Female copulatory organs were excised using sharpened needles and then transferred to lactic acid for examination under the microscope, after the fatty tissue dissolved, the vulvae were immersed in Hoyer's solution for imaging. The left male palp is shown unless otherwise indicated. All specimens are preserved in 75% ethanol. All measurements are taken in millimeters. Leg measurements are shown as: total length (femur, patella, tibia, metatarsus, tarsus). Leg podomeres were measured on their dorsal side. The distribution map was generated with ArcView GIS 3.2. References to figures in the cited papers are listed in lowercase (figure or figures); figures from this paper are noted with an initial capital (Figure or Figures).

Chelicerae were photographed with an FEI Quanta 450 environmental scanning electron microscope (SEM). Prior to examination, they were cleaned with an ultrasonicator and critical point dried with a Leica EM CPD300 automated critical -point dryer. Before critical point drying, these specimens were gradually dehydrated in increasing concentrations of ethanol for 24-48 hs. Dried specimens were then mounted sputter coated for 120s using a Leica EM SCD050 super cool sputter coater.

All specimens have been deposited in the Institute of Zoology, Chinese Academy of Sciences (IZCAS) in Beijing, China. Specimens were stored in 95% ethanol at -20 ℃. Total genomic DNA was extracted from legs of a single specimen. For six species we were able to obtain the DNA barcodes. The samples for *S. gaolingensis* Wu & Li **sp. nov.**, *S. huangniuensis* Wu & Li **sp. nov.**, *S. pulianensis* Wu & Li **sp. nov.**, were not extracted successfully. A partial fragment of the mitochondrial gene cytochrome oxidase subunit Ⅰ (*COI*) was amplified and sequenced following the protocol in Miller et al. (2010). Primers used in this study are: LCO1490 (5'-CWACAAAY­ CATARRGATA TTGG-3') and HCO-N-2198 (5'-TAAACTTCAGGGTGAC­ CAA AAAATCA-3') (Folmer et al., 1994). Voucher information and GenBank accession Nos. for all samples are listed in Table 1.

**Table 1:** GenBank accession Nos. for *COI* data obtained for this study

Species	GenBank accession No.	Collection localities
*Stedocys ludiyanensis* Wu & Li **sp. nov.**	KY197477	Ludiyan Cave, Guilin City, Guangxi, China
*Stedocys matuoensis* Wu & Li **sp. nov.**	KY197478	Matuo Cave, Longlin County, Baise City, Guangxi, China
*Stedocys shilinensis* Wu & Li **sp. nov.**	KY197479	Shilin Cave, Maogan Town, Baoting County, Hainan, China
*Stedocys xianrenensis* Wu & Li **sp. nov.**	KY197480	Xianren Cave, Longan County, Nanning City, Guangxi, China
*Stedocys xiangzhouensis* Wu & Li **sp. nov.**	KY197481	A cave without name, Xiangzhou County, Laibin City, Guangxi, China
*Stedocys zhaoi* Wu & Li **sp. nov.**	KY197482	A cave without name, Sai Yok District, Kanchanaburi, Thailand

**Taxonomy**

**Family Scytodidae Blackwall, 1864**

**Genus *Stedocys*Ono, 1995**

*Stedocys* Ono, 1995: 132. Labarque et al., 2009: 2. Type species by original designation: *Stedocys uenorum* Ono, 1995.

**Figure 1:**
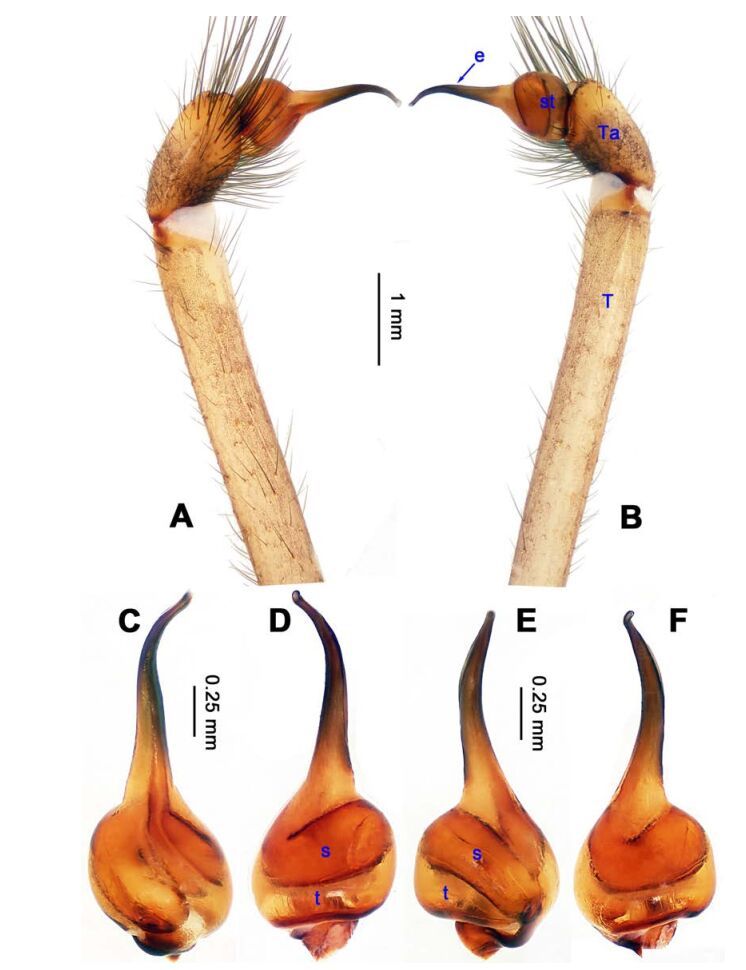
*Stedocys* *gaolingensis* sp. nov., holotype male

**Figure 4:**
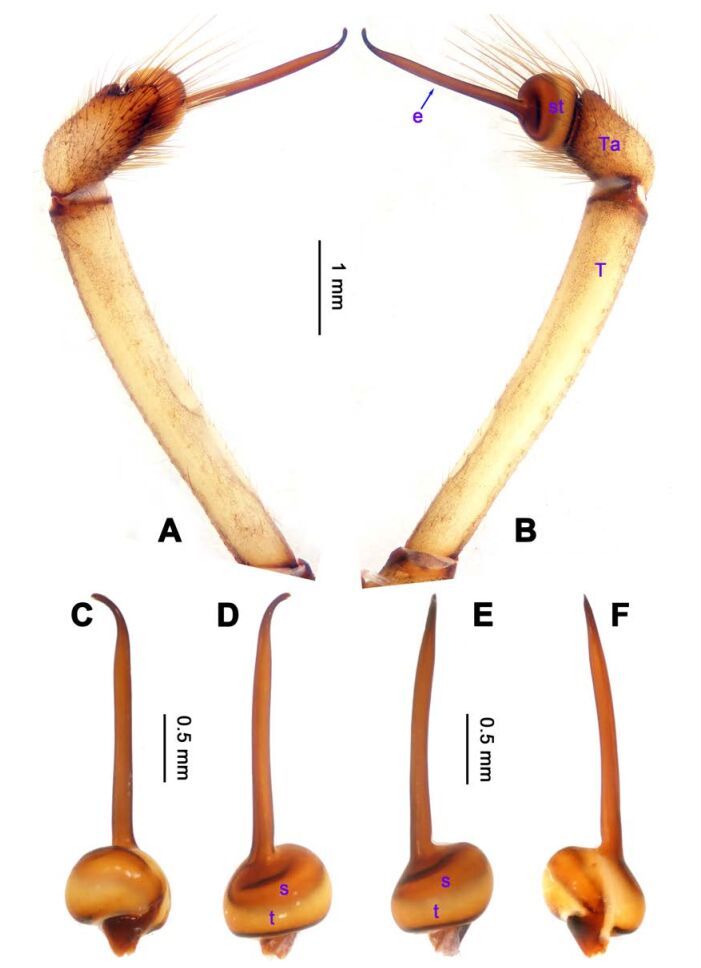
*Stedocys ludiyanensis* sp. nov., holotype male

**Figure 5:**
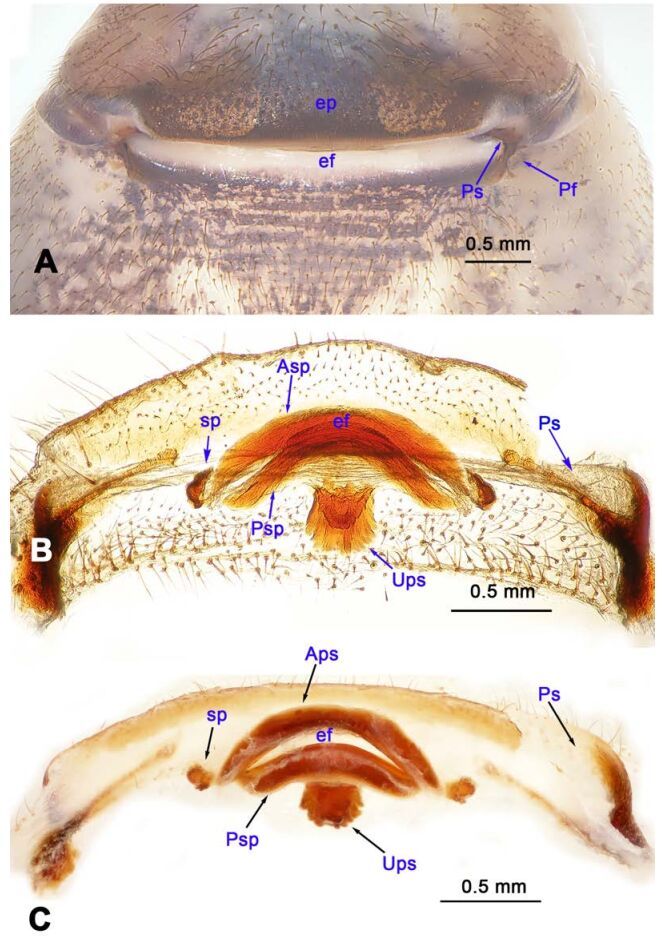
*Stedocys ludiyanensis* sp. nov., paratype female

**Figure 7:**
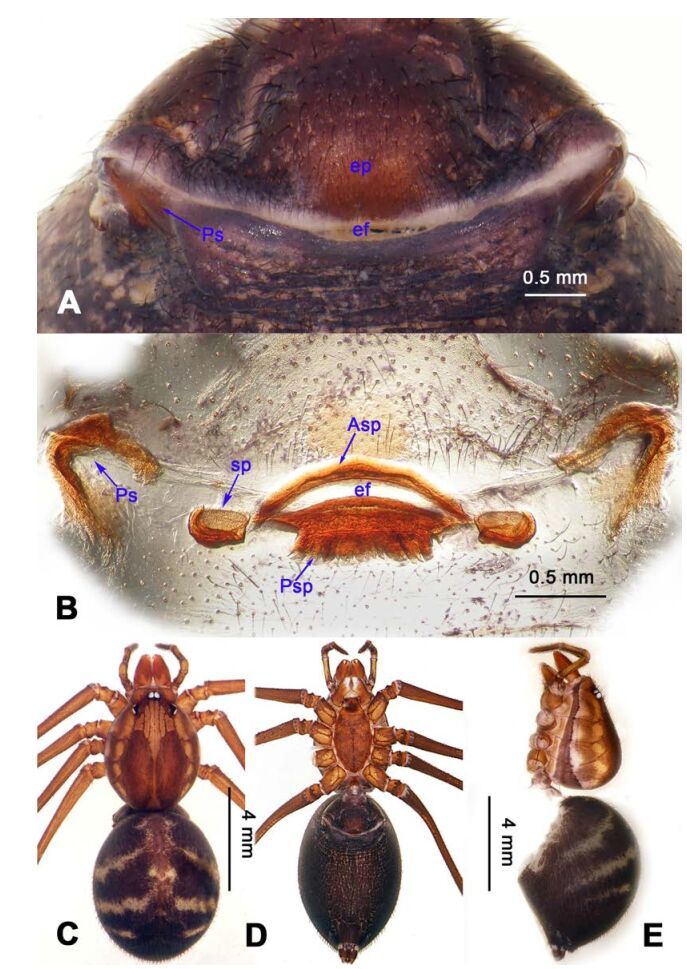
*Stedocys* *matuoensis* sp. nov., holotype female

**Figure 14:**
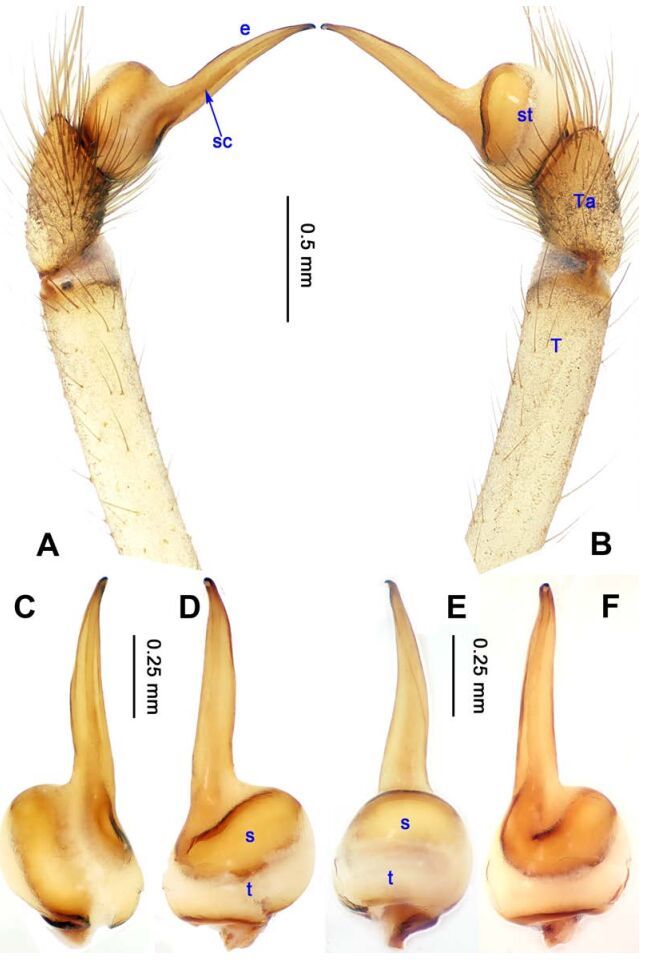
*Stedocys xiangzhouensis* sp. nov., holotype male

**Figure 15:**
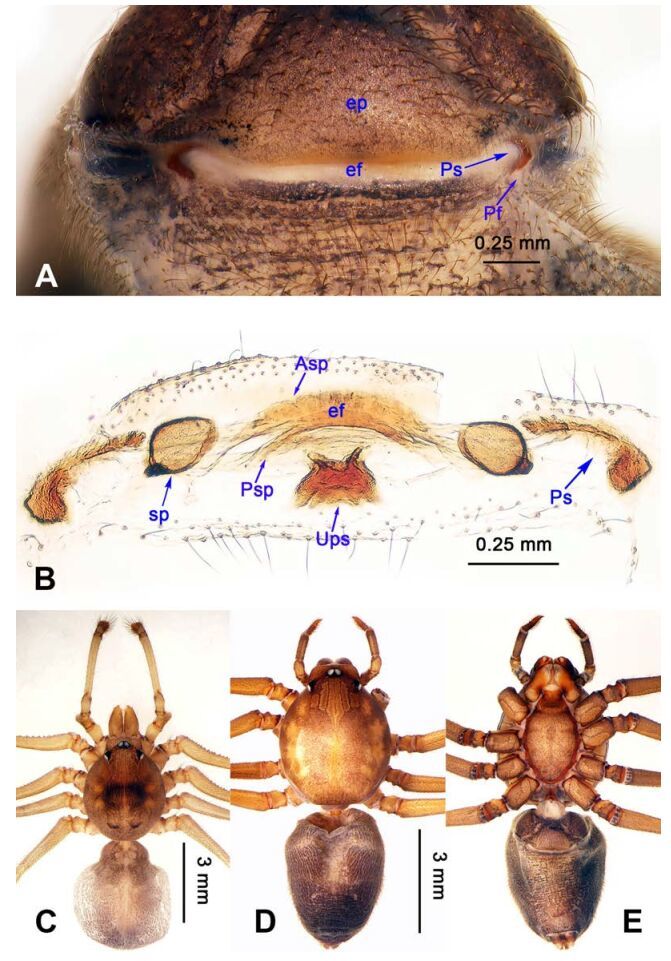
*Stedocys xiangzhouensis* sp. nov., holotype male (C) and paratype female (A-B, D-E)

**Figure 16:**
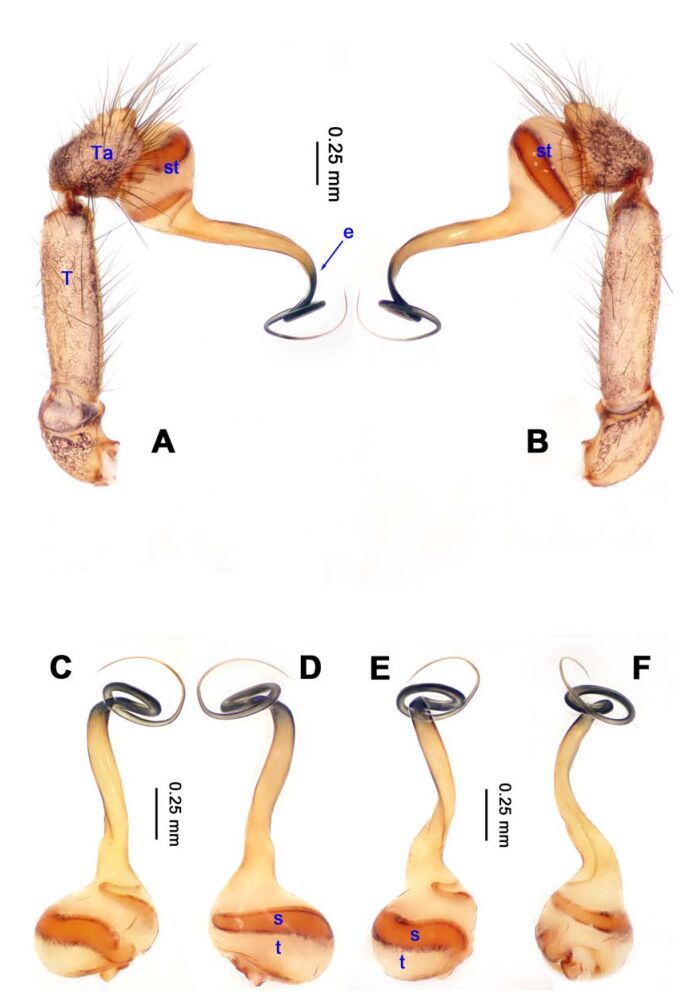
*Stedocys zhaoi* sp. nov., holotype male

**Figure 17:**
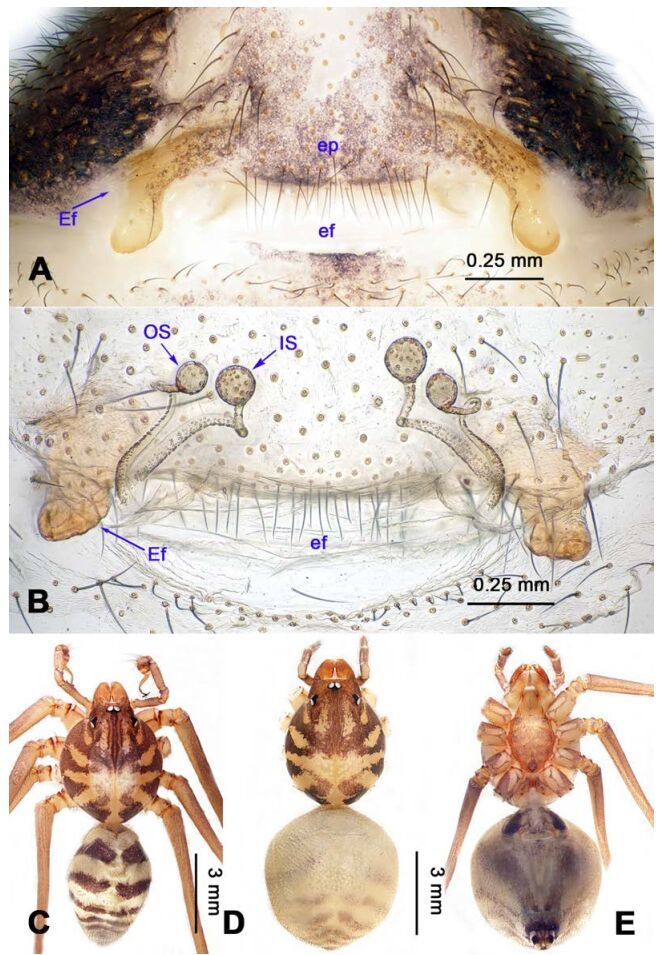
*Stedocys zhaoi* sp. nov., holotype male (C) and paratype female (A-B, D-E)

**Diagnosis.** Males of *Stedocys* are distinguished from other genera of Scytodidae by having a short cymbium, lacking an apical digitiform extension (present in other genera), an aciculate or coiled distal part of the embolus, and lacking a hyaline membrane in the tegular part of the palp (Figures 1A, 4A, 14A, 16A). Females can be distinguished from other genera by the presence of unpair sclerotized plate (Ups) (Figures 5B, 15B) and a slightly sclerotized post-(Figures 5A, 7A) or pre-epigastric foveae (Figure 17A-B).

**Figure 18:**
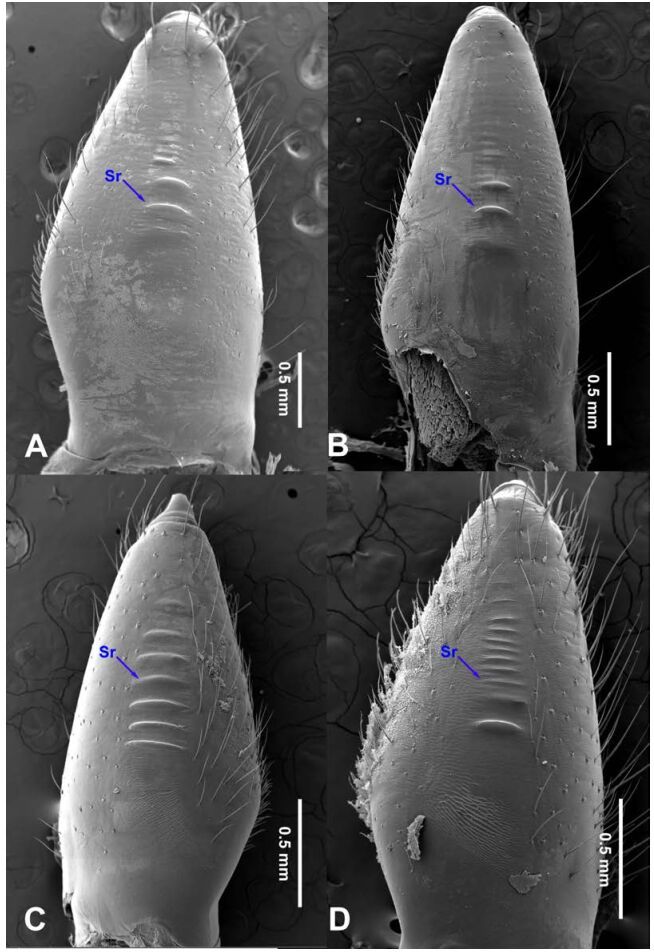
Chelicerae, ectal view

**Figure 19:**
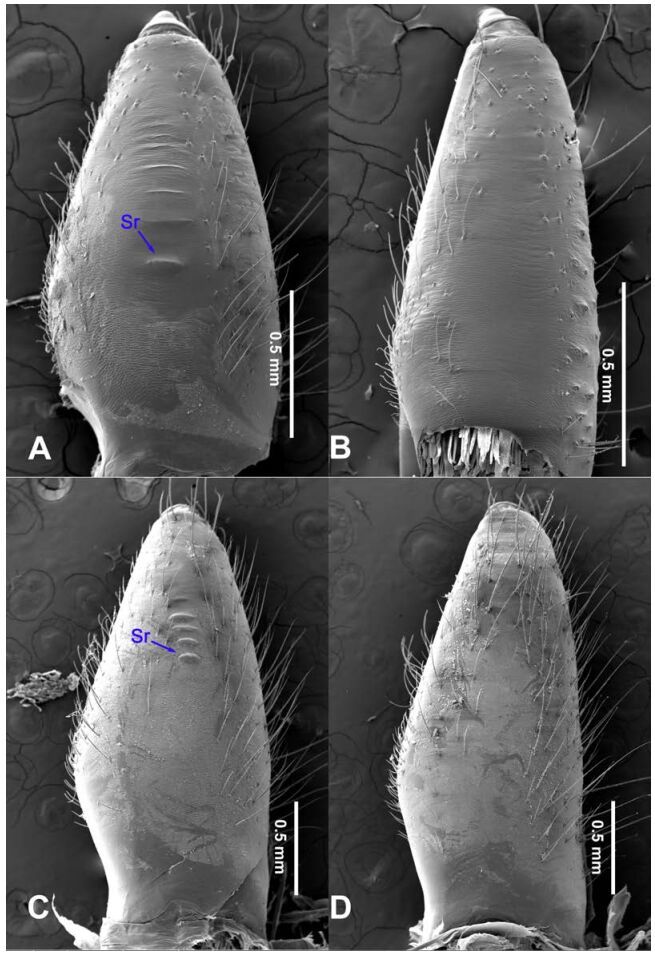
Chelicerae, ectal view

**Figure 20:**
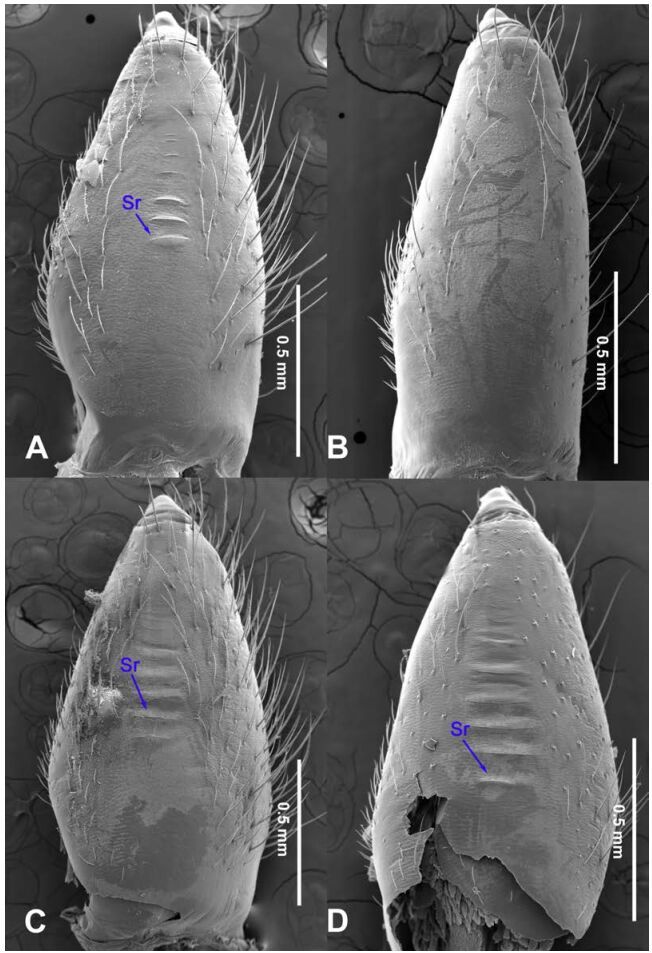
Chelicerae, ectal view

**Figure 21:**
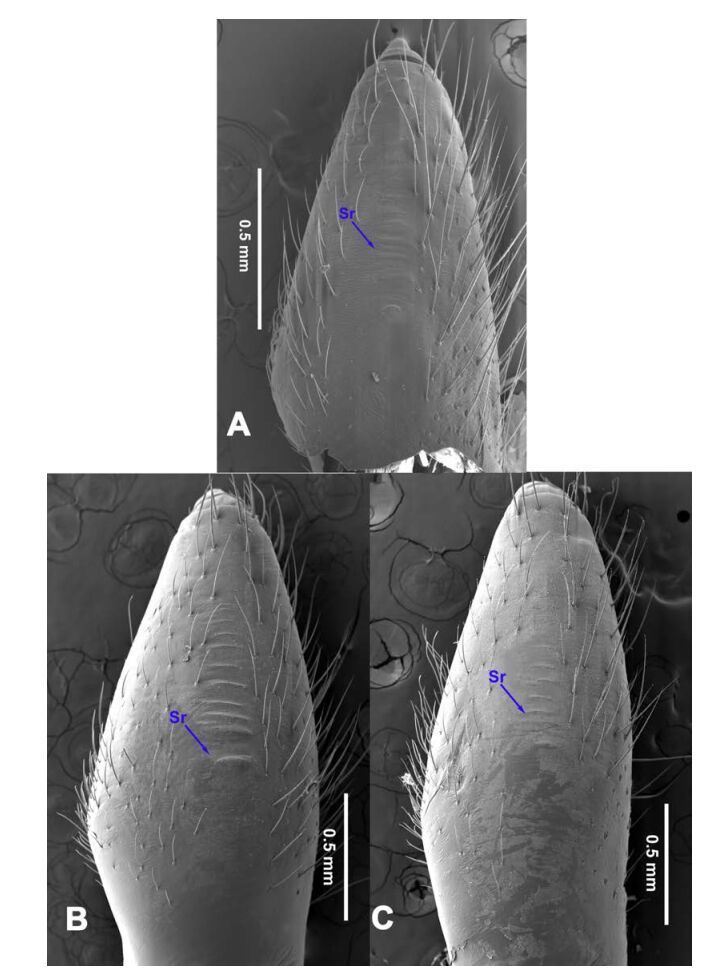
Chelicerae, ectal view

**Description.** Total length (males and females) 10.26-23.05. Prosoma longer than wide, posteriorly domed, thoracic furrow present in some species. Chelicerae sturdy and long compared to fangs, with a triangular cheliceral medial lamina separated from the paturon's margin by a deep depression, with setae distribted on the cheliceral lobe, and stridulatory ridges in all females (Figures 18-21). Leg formula Ⅰ-Ⅱ-Ⅳ-Ⅲ. All tarsi with well-developed onychia, and claw Ⅰ and Ⅱ with a bipectinate proclaw (see Labarque et al., 2009: 7, figures 21-24). Female palp with a pair of apical prolateral blunt macrosetae (see Labarque et al., 2009: 7, figures 27, 30). Colulus well defined, with developed posterior projection (see Labarque et al., 2009: 7, figures 31, 44, 46), if a feature isn't mentioned in a description, it is the same as that for the genus.

*Male palp*: Cymbium short, with short or no apical extension; embolus aciculate or coiled distally, without stylus in the apex of the bulb.

*Vulva*: Atypical for the family, with a single sclerotized plate (Figures 5B, 15B), and slightly sclerotized post-or pre-epigastric foveae, with one or two pairs of spermathecae, arising from the epigastric furrow.

**Natural history.** Specimens were found in humid karst caves hanging on webs in the aphotic zone, far from the entrance of the cave, or at the low light zone of the cave.

**Distribution.** Southeast Asia (South China, Malaysia, Thailand).

***Stedocys gaolingensis* Wu & Li sp. nov.**

Figures 1-2, 18A-B

**Figure 2:**
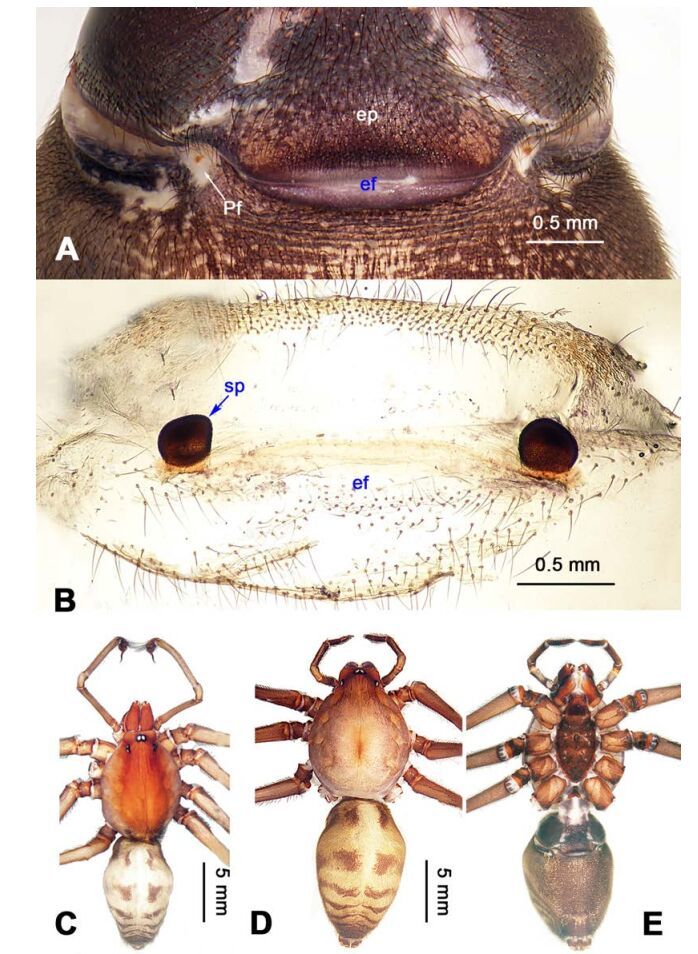
*Stedocys* *gaolingensis* sp. nov., holotype male (C) and paratype female (A-B, D-E)

**Type material. Holotype:** ♂, Cave No. 2 (N24°05.711′, E108°04.954′, 175 m a.s.l.), Longguangshangtun, Longchi Village, Gaoling Town, Duan County, Hechi City, Guangxi, China, 06.03.2016, X. Zhang and Z. Chen. **Paratype**: 1♀, same data as holotype.

**Etymology.** The specific name refers to the type locality; adjective.

**Diagnosis.** The male can be easily distinguished from the other species by having the cymbium almost 2 times longer than the tegular part of the bulb (Figure 1A-B), by the embolus being subequal to the palpal tarsus in length, and the embolus slightly bent ventrally; females are easily distinguished from other species by the large, round spermathecae (Figure 2B).

**Description. Male** **(holotype):** Total length 18.82; carapace 9.05 long, 6.35 wide; chelicerae 2.10 long, labium 2.15 long, 1.15 wide, sternum 2.85 long, 1.05 wide; opisthosoma 9.52 long, 5.57 wide; . Leg Ⅰ: 100.88 (29.52, 2.75, 28.25, 37.11, 3.25), leg Ⅱ: -(27.26, 2.64, 25.66, -, -), leg Ⅲ: 55.78 (17.12, 2.41, 15.55, 18.25, 2.45), leg Ⅳ: 73.73 (21.75, 2.75, 22.25, 23.93, 3.05), palp: 14.39 (6.35, 1.52, 5.27, -, 1.25). Carapace pale reddish-brown, with transverse brown stripes and longitudinal bands marginally, covered with dense hairs posteriorly, thoracic furrow shallow (Figure 2C). Eye sizes: PME 0.38; ALE 0.35; PLE 0.35. Chelicerae with 2 conspicuously spaced stridulatory ridges (Figure 18B), fangs, endites, labium sternum and colulus follow the generic pattern. Habitus as in Figure 2C. Legs yellowish-brown, very long, five times longer than the body length, and slender (Figure 2C). Opisthosoma whitish-yellow dorsally, the first half with two irregular pairs of brown marks extending laterally and four pairs of chevron-like marks on the posterior half. Palp as in Figure 1A-F; tip of cymbium covered with dense cluster of setae, embolus slightly curved, embolus tip apically blunt, bulb 1.55 long, elongated (Figure 1B).

**Female:** Total length 23.05, carapace 11.05 long, 8.25 wide; chelicerae 2.10 long, sternum 5.25 long, 3.55 wide; labium 2.62 long, 1.35 wide; opisthosoma 12.02 long, 7.75 wide. Leg Ⅰ: 89.30 (25.52, 2.92, 25.65, 31.75, 3.46), leg Ⅱ: 78.18 (22.75, 2.82, 22.82, 26.54, 3.25), leg Ⅲ: 54.53 (16.55, 2.85, 15.35, 17.15, 2.63), leg Ⅳ: 72.69 (22.16, 3.05, 21.98, 22.75, 2.75), palp: 9.47 (3.04, 1.32, 2.49, -, 2.62). Prosoma with brown radial stripes and reddish markings marginally, highest at the center, covered by slender hairs posteriorly, thoracic furrow conspicuous (Figure 2D), chelicerae with 3 stridulatory ridges mesally (Figure 18A). Eye diameters: PME 0.44; ALE 0.38; PLE 0.38. Other characters similar to male except for the color: the female has a more yellow carapace and a browner opisthosoma and legs. Habitus as in Figure 2D-E. Vulva (Figure 2B) with only one pair of oval receptacles with an interdistance of 1.53.

**Figure 22:**
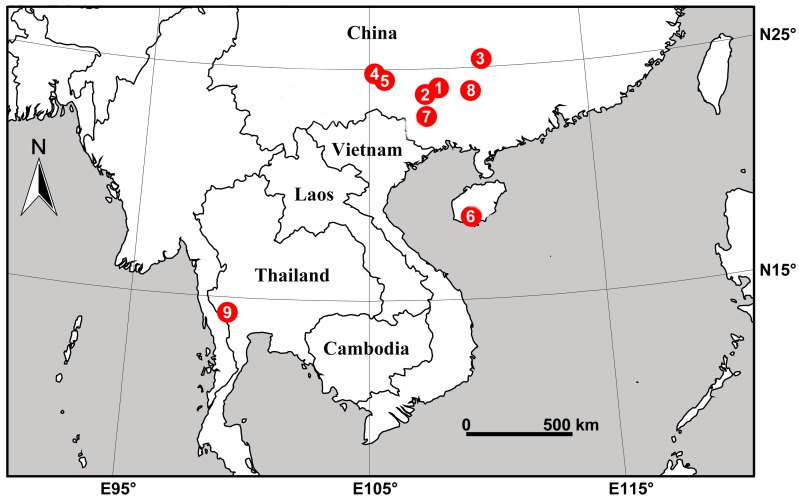
Distribution records of new *Stedocys* species from China and Thailand

**Distribution.** Known only from type locality in Guangxi, China (Figure 22).

**Natural History.** The species was found in the aphotic zone, far from the entrance of the long, humid cave.

***Stedocys huangniuensis* Wu & Li sp. nov.**

Figures 3, 18C

**Figure 3:**
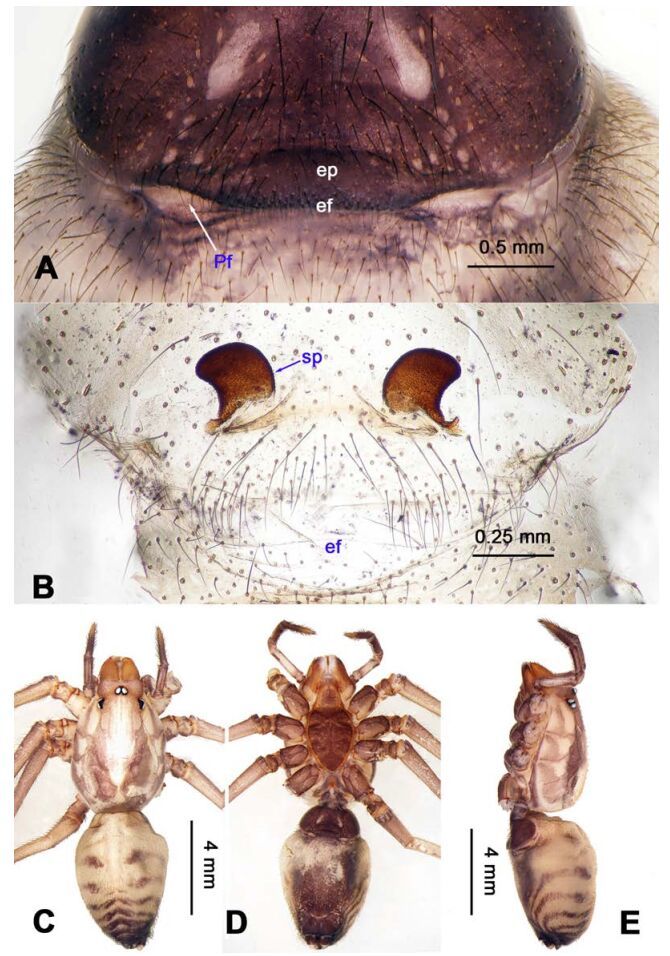
*Stedocys* *huangniuensis* sp. nov., holotype female

**Type material. Holotype:** ♀, Huangniu Cave (N23°55.120′, E107°37.479′, 175 m a.s.l.), Damo Village, Jiangnan Town, Dahua County, Hechi City, Guangxi, China, 23.09.2015, J. Wu and Z. Chen leg. **Paratypes:** 5♀, same data as holotype; 3♀, same locality, 08.03.2007, J. Liu and Y. Lin.

**Etymology.** The specific name refers to the type locality; adjective.

**Diagnosis.** The female resembles that of *S. gaolingensis* **sp. nov.** (Figures 1-2) in having a pair of receptacles in the uterus externus, but can be easily distinguished from other species by the non-oval spermathecae and the relatively short distance between spermathecae (Figure 3B).

**Description. Female (holotype):** Total length 12.77, prosoma 6.27 long, 3.76 wide, opisthosoma 6.25 long, 3.75 wide, labium 2.48 long, 1.75 wide, sternum 2.77 long, chelicerae 1.15, 2.25 wide. Leg Ⅰ: 47.02 (14.75, 1.77, 10.25, 17.28, 2.97), leg Ⅱ: 40.97 (12.15, 1.75, 11.77, 12.76, 2.54), leg Ⅲ: 30.54 (8.75, 1.54, 8.75, 9.25, 2.25), leg Ⅳ: 41.07 (10.47, 1.75, 12.54, 13.78, 2.53), palp: 7.23 (2.37, 0.98, 1.73, -, 2.15). Carapace whitish-brown with faint brown radial stripes and longitudinal bands laterally (Figure 3E), highest at the center, ocular area covered with few hairs around eyes, thoracic furrow absent (Figure 3C). Eye diameters: PME 0.34; ALE 0.29; PLE 0.28. Chelicerae with 7 stridulatory ridges (Figure 18C), fangs, endites, labium sternum and colulus follow generic pattern. (Figure 3D). Habitus as in Figure 3C-E. Legs yellowish, without rings or spines. Opisthosoma brownish-yellow dorsally, anterior half with 4 irregular spots and 5 pairs of chevron-like marks on posterior half. Colulus trapeziform. Vulva (Figure 3B) with a pair of oval spermathecae, and the interdistance between spermathecae is 0.33.

**Male:** Unknown

**Distribution.** Known only from type locality in Guangxi, China (Figure 22).

**Natural History.** The female was found in the aphotic zone, deep inside the long, humid cave, hanging on its web with many immature spiders.

***Stedocys ludiyanensis* Wu & Li sp. nov.**

Figures 4-6, 21B-C

**Figure 6:**
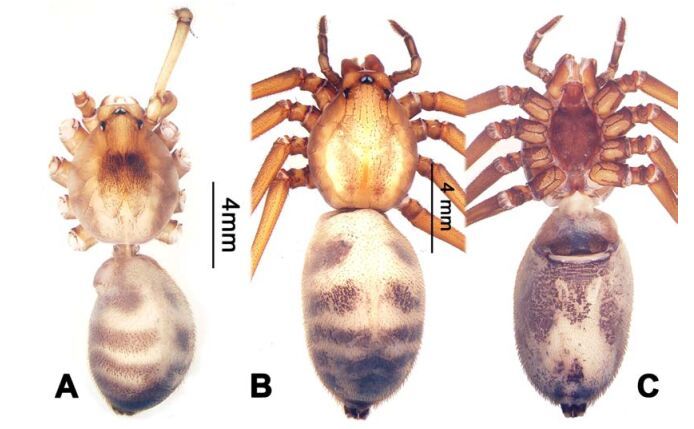
*Stedocys ludiyanensis* sp. nov., holotype male (A) and paratype female (B, C), habitus

**Type material. Holotype:** ♂, Ludiyan Cave (N25°18.527′, E110°15.833′, 164 m a.s.l.), Guilin City, Guangxi, China, 07.10.2010, X. Wang and L. Lin. **Paratypes:** 8♀, same data as holotype.

**Etymology.** The specific name refers to the type locality; adjective.

**Diagnosis.** The male can be easily distinguished from all known congeners in *Stedocys* by having the tarsus 2 times longer than the basal portion of the bulb, and by the long, slender and straight embolus that is 1.5 times longer than the tarsus (Figure 4A-B). The female can be distinguished by the relatively small spermathecae beside the uterus externus, by the having the uterus externus enclosed by the curved anterior plate and posterior plate, by the postepigastric fovea with postgastral sulci, and by the unpaired posterior sclerotization underneath the posterior plate (Figure 5B-C).

**Description. Male** **(holotype):** Total length 16.10, carapace 7.45 long, 5.85 wide, labium 1.55 long, 1.25 wide, sternum 3.58 long, opisthosoma 8.13 long, 5.65 wide, 2.75 wide. Leg Ⅰ: 87.26 (23.33, 2.13, 28.55, 30.62, 2.63), leg Ⅱ: 67.93 (19.05, 2.25, 19.74, 24.25, 2.64), leg Ⅲ: 37.52 (12.11, 2.14, 11.53, 9.59, 2.15), leg Ⅳ: 48.67 (16.05, 2.15, 16.48, 11.35, 2.64), palp: 9.85 (3.62, 1.35, 3.83, -, 1.05). Carapace pale brownish yellow, with brown radiating stripes and reddish bands marginally, highest at the middle posteriorly, with an arcuate appearance from the dome to the clypeus in lateral view, covered by dense serrate hairs posteriorly, no thoracic furrow (Figure 6A). Six eyes follow generic pattern. Eye diameters: PME 0.36; ALE 0.35; PLE 0.35. Chelicerae with seven stridulatory ridges mesally (Figure 21C), fangs, endites, labium sternum and colulus follow generic pattern. Habitus as in Figure 6A. Legs yellowish, without spines or annulated marks, claws follow generic pattern. Opisthosoma whitish dorsally, the first half with two pairs of irregular marks extending laterally and three pairs of chevron-like marks on the posterior half. Spinnerets short, follow generic pattern; colulus oval, can be easily observed. Palps as in Figure 4A-F; palp blunt apically, the fusion of subtegulum and tegulum shorter than tarsus; the apex of tarsus covered with a dense cluster of hair, the apex of the embolus gradually curved, bulb implanted prolaterally in tarsus (Figure 4B).

**Female:** Total length 14.73, chelicerae 1.51, with a long series of 10 spaced stridulatory ridges ectal mesally (Figure 21B), prosoma 6.25 long, 4.75 wide, opisthosoma 8.48 long, 5.53 wide, labium 1.25 long, 1.15 wide, sternum 3.05 long, 2.25 wide. Leg Ⅰ: 43.86 (12.65, 1.67, 12.94, 14.25, 2.35), leg Ⅱ: 37.52 (11.05, 1.66, 10.95, 11.75, 2.11), leg Ⅲ: 26.57 (8.05, 1.67, 7.35, 7.85, 1.65), leg Ⅳ: 35.50 (10.48, 1.75, 10.63, 10.89, 1.75), palp: 5.74 (1.65, 0.63, 1.51, -, 1.95). Prosoma with brown radiating stripes and a longitudinal pattern, highest at the center, with few hairs, and a conspicuous thoracic furrow (Figure 6B). Eye diameters: PME 0.44; ALE 0.35; PLE 0.41. Other characters similar to male except for the more yellow opisthosoma and browner legs. Habitus as in Figure 6B-C. Vulva (Figure 5B-C) contains a pair of spermathecae with conspicious postion ridge.

**Distribution.** Known only from type locality in Guangxi, China (Figure 22).

**Natural History.** The male was found in the aphotic zone, far from the entrance of the long, humid cave, hanging on its web. The females were found both closer to and far from the entrance of the long, humid cave.

***Stedocys matuoensis* Wu & Li sp. nov.**

Figures 7, 18D

**Type material. Holotype:** ♀, Matuo Cave (N24°44.511′, E105°22.473′, 724 m a.s.l.), Lengshui Village, Xinzhou Town, Longlin County, Baise City, Guangxi, China, 30.09.2015, J. Wu and Z. Chen. **Paratypes:** 16♀, same data as holotype; 6♀, same locality, 02.01.2011, Z. Chen and Z. Zha.

**Etymology.** The specific name refers to the type locality; adjective.

**Diagnosis.** The female can be easily distinguished from all known congeners of *Stedocys* by the curved, broad slit formed by the anterior and posterior sclerotized plate between the oval spermathecae and the arcuate postgastral sulci beside the spermathecae in the vulvae (Figure 7B).

**Description. Female** **(holotype):** Total length 12.78, carapace 5.75 long, 3.75 wide, opisthosoma 6.53 long, 5.05 wide, chelicerae 1.65, labium 1.15 long, 0.65 wide, sternum 2.15 long, 1.75 wide. Leg Ⅰ: 32.93 (10.25, 1.27, 10.05, 10.11, 1.25), leg Ⅱ: 28.71 (8.57, 1.26, 8.48, 8.55, 1.85), leg Ⅲ: 21.96 (6.05, 1.05, 6.75, 6.47, 1.64), leg Ⅳ: 26.85 (8.25, 1.05, 8.05, 7.75, 1.75), palp: 4.25 (1.33, 0.52, 1.05, -, 1.35). Carapace chestnut brown, with deep brown radiating stripes and one pair of oval marks (Figure 7C), highest at the center, with few hairs around eyes, andlacking thoracic furrow (Figure 7C). Six eyes follow the generic pattern. Eye diameters: PME 0.44; ALE 0.38; PLE 0.38. Chelicerae with a long series of 11 conspicuous, densely spaced stridulatory ridges ectally (Figure 18D). Habitus as in Figure 7C-E. Legs brown, without spines and annulated marks.Opisthosoma black dorsally, with three pairs of transverse grey bands markings. The spermathecae interdistance is 1.25 (Figure 7B).

**Male:** Unknown

**Distribution.** Known only from the type locality in Guangxi, China (Figure 22).

**Natural History.** The females were found in the low light and aphotic zone, both near to and far from the entrance of the long, humid cave, hanging on irregular webs with several immature spiders. There were many mature female species in the cave but no males were found after three hours of collecting by two people.

***Stedocys pulianensis* Wu & Li sp. nov.**

Figures 8-9, 21A

**Figure 8:**
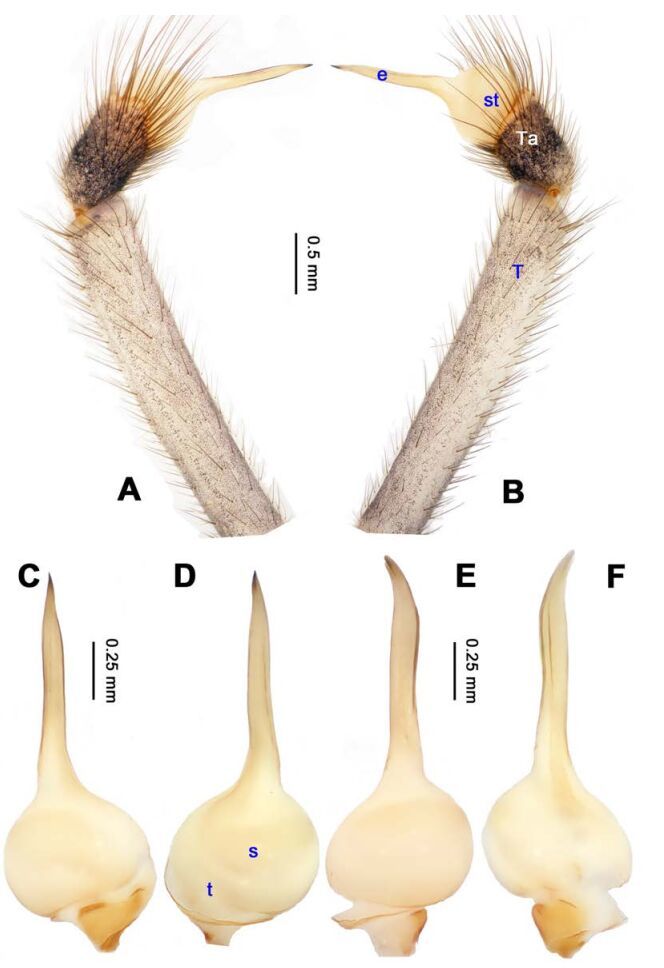
*Stedocys* *pulianensis* sp. nov., holotype male

**Figure 9:**
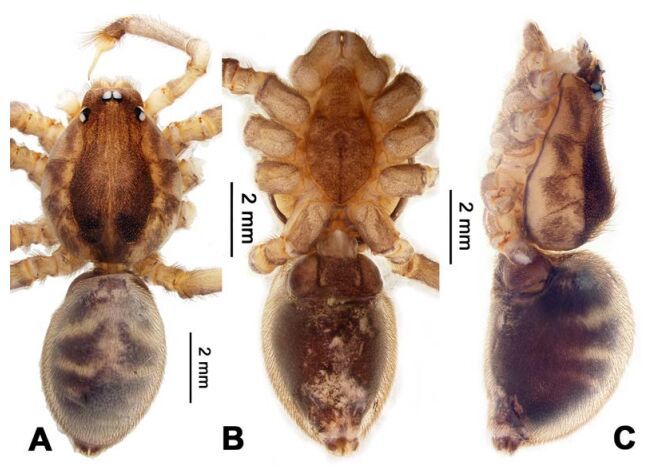
*Stedocys pulianensis* sp. nov., holotype male, habitus

**Type material. Holotype:** ♂, Pulian Cave (N24°51.656′, E105°65.948′, 975 m a.s.l.), Tongxiang Village, Dongxiang, Pingtang Town, Tianyang County, Baise City, Guangxi, China, 12.08.2011, X. Wang.

**Other material examined.** 1♀, immature, same data as holotype.

**Etymology.** The specific name refers to the type locality; adjective.

**Diagnosis.** The male species can be easily distinguished from all known congeners in *Stedocys* by the cylindrical tarsus, subequal to or longer than the basal portion of the bulb, and by the dense hair around the tarsus and tibia (Figure 8A-B).

**Description. Male (holotype):** Total length 10.26, carapace 5.15 long, 4.22 wide, opisthosoma 5.11 long, 3.95 wide, chelicerae 1.32, labium 1.25 long, 0.83 wide, sternum 2.25 long, 1.82 wide. Leg Ⅰ: 53.07 (16.25, 1.65, 16.05, 16.49, 2.63), leg Ⅱ: 46.35 (14.15, 1.65, 13.45, 14.75, 2.35), leg Ⅲ: 29.92 (9.02, 1.55, 8.75, 8.75, 1.85), leg Ⅳ: 41.14 (12.25, 1.55, 12.54, 12.35, 2.45), palp: 7.83 (3.11, 1.02, 3.05, -, 0.65). Carapace chestnut brown, with yellowish-brown vertical stripes and reddish bands marginally, highest at center, with a few hairs posteriorly, and no thoracic furrow (Figure 9A). Six eyes follow the generic pattern. Eye diameters: PME 0.35; ALE 0.32; PLE 0.32. Chelicerae with a series of spaced inconspicuous stridulatory ridges ectally (Figure 21A), fangs, endites, labium, sternum, and spinnerets follow the generic pattern. Habitus as in Figure 9A-C. Legs brown, without rings or spines. Opisthosoma brown dorsally, with two whitish-brown transverse bands. Colulus triangular. Palps as in Figure 8A-F; palp with a long embolus, apically aciculate.

**Distribution.** Known only from type locality in Guangxi, China (Figure 22).

**Natural History.** The male was found in the aphotic zone. Far from the entrance of the humid cave, hanging on its web.

***Stedocys shilinensis* Wu & Li sp. nov.**

**Figure 10:**
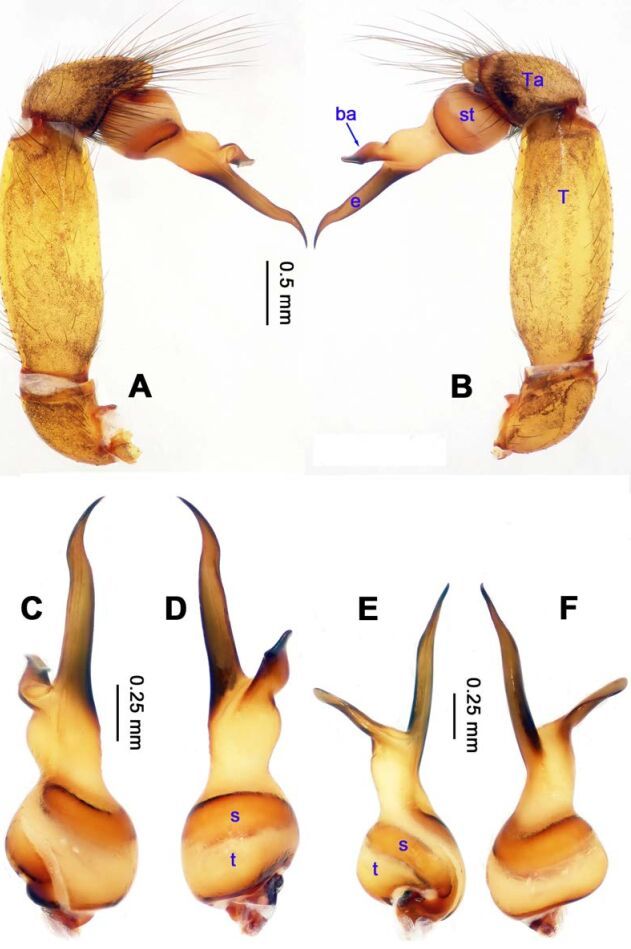
*Stedocy shilinensis* sp. nov., holotype male

**Figure 11:**
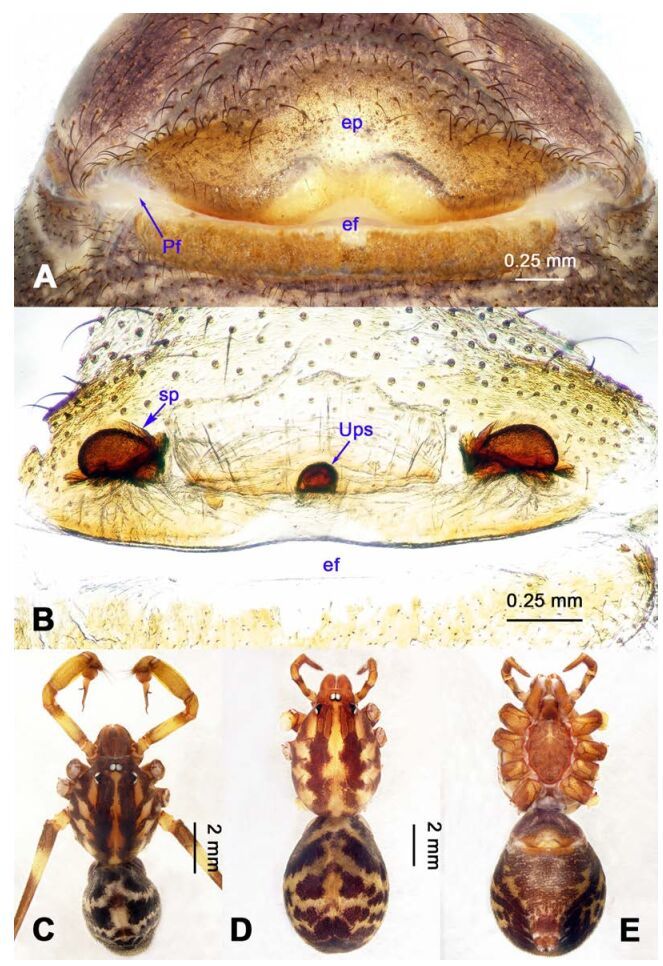
*Stedocy shilinensis* sp. nov., holotype male (C) and paratype female (A-B, D-E)

Figures 10-11, 19A-B

**Type material. Holotype:** ♂, Shilin Cave (N18°35.861′, E109°25.611′, 616 m a.s.l.), Xian An Stone Forest, Maogan Town, Baoting County, Hainan, China, 25.06.2014, X. Wang and F. Li.

**Paratypes:** 4♀, same data as holotype.

**Etymology.** The specific name refers to the type locality; adjective.

**Diagnosis.** The male can be easily distinguished from all known congeners by having an apophysis on the bulb of male palp (Figure 10A-B) and the female genitalia contains a simple median unpaired sclerotized plate (Figure 11B).

**Description. Male (holotype):** Total length 8.67, carapace 4.35 long, 3.51 wide, opisthosoma 4.32 long, 3.25 wide, chelicerae 1.14, labium 1.25 long, 0.96 wide, sternum 2.48 long, 1.75 wide. Legs Ⅰ and Ⅳ missing, leg Ⅱ: 39.67 (11.46, 1.35, 12.35, 11.98, 2.53), leg Ⅲ: 25.21 (7.27, 1.39, 7.05, 7.75, 1.75). Palp: 6.37 (2.47, 0.87, 1.98, -, 1.05). Carapace orange with bilaterally brown radiated stripes pattern and two longitudinal bands, highest at center, covered with scarce hairs, no thoracic furrow (Figure 11C). Eye diameters: PME 0.29; ALE 0.31; PLE 0.32. Chelicerae without obvious spaced stridulatory ridges (Figure 19B), fangs, endites, labium, sternum, and spinnerets follow the generic pattern. Habitus as in Figure 11C. Legs yellowish-brown, long and stout, with brown annulations, claws follow generic pattern. Opisthosoma whitish-brown dorsally, with fragmented, irregular black stripes; colulus triangular. Palps as in Figure 10A-F; palp with an apophysis on the bulb, embolus apically aciculate, the basal portion of the bulb almost subequal in length to tarsus, the apex of tarsus with a dense cluster of hairs, visible in prolateral view (Figure 10B).

**Female:** Total length 9.87, carapace 4.15 long, 3.25 wide, opisthosoma 5.72 long, 3.75 wide, chelicerae 1.15, labium 1.13 long, 0.75 wide, sternum 2.48 long, 1.88 wide. Leg Ⅰ: 40.11 (11.53, 1.53, 12.25, 12.15, 2.65), leg Ⅱ: 31.37 (9.25, 1.45, 9.75, 8.77, 2.15), leg Ⅲ: 22.87 (6.95, 1.37, 6.25, 6.55, 1.75), leg Ⅳ: 32.14 (9.05, 1.35, 9.84, 9.75, 2.15), palp: 4.68 (1.38, 0.64, 1.13, -, 1.53). Carapace highest at the center, with a few hairs, color as in male, slightly lighter, lacking thoracic furrow (Figure 11D). Six eyes follow the generic pattern. Eye diameters: PME 0.26; ALE 0.29; PLE 0.30; chelicerae with 7 conspicuous spaced stridulatory ridges ectally (Figure 19A). Other characters similar to those of male, and palps and claws follow the generic pattern. Habitus as in Figure 11D-E. The spermathecae interdistance is 1.13 (Figure 11B).

**Distribution.** Known only from type locality in Hainan, China (Figure 22).

**Natural History.** The species was found in the aphotic zone, far from the entrance of the long, humid cave, hanging on its web.

***Stedocys xianrenensis* Wu & Li sp. nov.**

**Figure 12:**
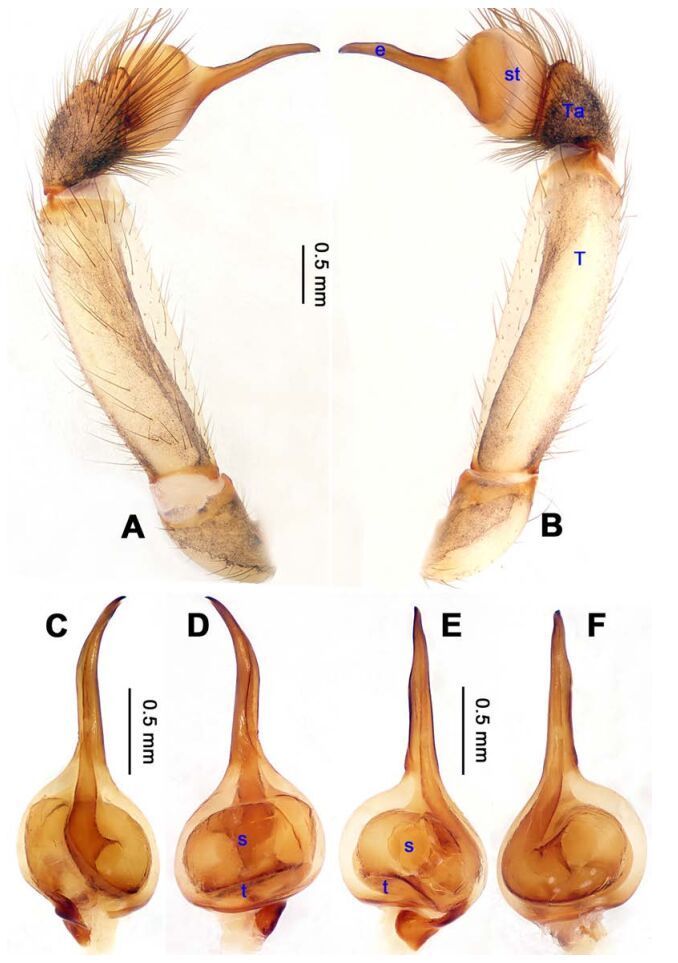
*Stedocys* *xianrenensis* sp. nov., holotype male

**Figure 13:**
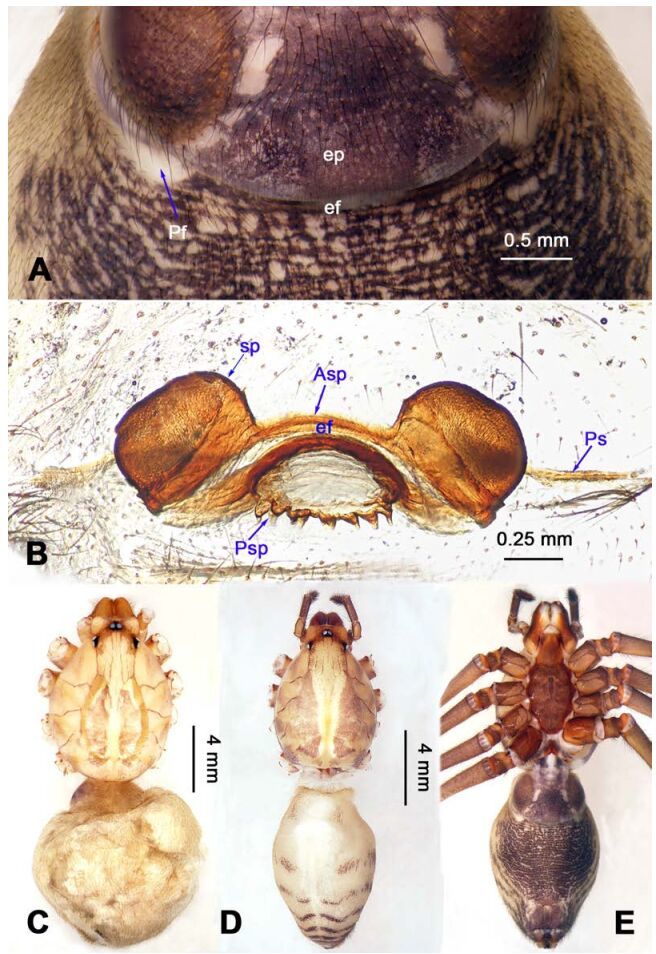
*Stedocys* *xianrenensis* sp. nov., holotype male (C) and paratype female (A-B, D-E)

Figures 12-13, 19C-D

**Type material. Holotype :** ♂, Xianren Cave (N22°57.535′, E107°36.948′, 241 m a.s.l.), Mount Longhu Nature Reserve, Longan County, Nanning City, Guangxi, China, 18.10.2010, X. Wang and L. Lin. **Paratypes:** 4♀, same data as holotype; 10♀, same locality, 20.09.2015, J. Wu and Z. Chen.

**Etymology.** The specific name refers to the type locality; adjective.

**Diagnosis.** The male can be easily distinguished from all known congeners by the relatively short tarsus that has a small extension, by the tarsus being subequal or smaller than the oval basal portion of the bulb, and by the triangular tarsus in retrolateral view (Figure 12A-B). The female can be easily distinguished from other species by the very large pair of round spermathecae (Figure 13B).

**Description. Male (holotype):** Total length 17.75, carapace 8.75 long, 6.50 wide, opisthosoma 8.75 long, 9.50 wide, chelicerae 1.85, labium 1.65 long, 1.25 wide, sternum 4.15 long, 3.10 wide. Leg Ⅰ: 89.91 (25.15, 2.65, 26.16, 32.80, 3.15), leg Ⅱ: 82.55 (23.25, 2.65, 24.15, 29.75, 2.75), leg Ⅲ: 51.98 (15.30, 2.50, 14.95, 16.98, 2.25), leg Ⅳ: 69.21 (20.17, 2.59, 21.15, 22.65, 2.65), palp: 9.09 (3.49, 1.35, 3.10, -, 1.15). Carapace pale yellow, with brown radiating stripes and reddish bands marginally, highest at the center, covered by slender hairs posteriorly, and with a shallow thoracic furrow (Figure 13C). Eye diameters: PME 0.33; ALE 0.37; PLE 0.35. Chelicerae without obvious stridulatory ridges (Figure 19D), and fangs, endites, labium, sternum, and spinnerets follow the generic pattern. Habitus as in Figure 13C. Legs yellowish-brown, long and slender, without rings and spines. Opisthosoma damaged. colulus triangular, conspicuous. Palps as in Figure 12A-F; palp with a long embolus, apically aciculate, slightly subdistally curved; the apex of the tarsus with dense cluster of hairs.

**Female:** Total length 17.88, prosoma 8.17 long, 6.15 wide, carapace 9.26 long, 6.15 wide, chelicerae 1.65, labium 1.75 long, 1.15 wide, sternum 3.65 long, 2.47 wide. Leg Ⅰ: 64.87 (19.55, 2.15, 18.75, 21.78, 2.64), leg Ⅱ: 56.62 (16.56, 2.15, 16.48, 18.88, 2.55), leg Ⅲ: 39.67 (11.47, 2.15, 11.15, 12.65, 2.25), leg Ⅳ: 53.49 (16.15, 2.15, 15.47, 17.15, 2.57), palp: 6.79 (2.15, 0.85, 1.45, -, 2.34). Prosoma with deep brown radiating stripes and reddish bands marginally, highest at the center, with slender hairs, and a conspicuous thoracic furrow (Figure 13D). Chelicerae with 5 conspicuous stridulatory ridges ectally (Figure 19C). Carapace whitish dorsally, the first half with a pair of brown spots and five chevron-like markings at the base (Figure 13D). Eye diameters: PME 0.30; ALE 0.32; PLE 0.35. Other characters similar to those of male except for the color which is more yellow in female. Habitus as in Figure 13D-E. Legs without rings and spines, and claws and spinnerets follow the generic pattern; colulus oval, conspicuous. Vulva (Figure 13B) contains a structure resembling the medusa stage of a jellyfish, made by posterior sclerotized plate.

**Distribution.** Known only from type locality in Guangxi, China (Figure 22).

**Natural History.** The species was found in the aphotic zone, far from the entrance of the long, humid cave, hanging on its web.

***Stedocys xiangzhouensis* Wu & Li sp. nov.**

Figures 14-15, 20A-B

**Type material. Holotype:** ♂, unnamed cave (N23°57.278′, E109°39.696′, 114 m a.s.l.), hill behind factory of man-made board, Xiangzhou County, Laibin City, Guangxi, China, 10.10.2010, X. Wang and L. Lin. **Paratypes:** 12♀, same data as holotype.

**Etymology.** The specific name refers to the type locality; adjective.

**Diagnosis.** The male can be easily distinguished from all known congeners by having the palpal tarsus a little longer than the basal portion of the bulb, by the long, stout and straight embolus that is 1.5 times as long as the basal portion of the bulb (Figure 14A-B). The female resembles *S. ludiyanensis* Wu & Li **sp. nov.** (Figures 4-6) in having a pair spermathecae beside the uterus externus, by the shape, enclosed by the curved anterior plate and posterior plate, by the postepigastric fovea with postgastral sulci, and by the unpaired posterior sclerotization beneath the posterior sclerotized plate. Additionally, it can be easily distinguished by the large oval spermathecae beside the uterus externus and the trapezoidal unpaired posterior sclerotized plate (Figure 15B).

**Description. Male (holotype):** Total length 7.80, carapace 3.75 long, 3.15 wide, opisthosoma 4.05 long, 3.35 wide, chelicerae 1.26, labium 1.05 long, 0.65 wide, sternum 1.75 long, 1.65 wide. Leg Ⅰ: 53.95 (19.75, 1.15, 19.95, 11.35, 1.75), leg Ⅱ: 30.08 (8.95, 1.18, 8.65, 9.65, 1.65), leg Ⅲ: 18.62 (5.69, 1.15, 5.15, 5.58, 1.05), leg Ⅳ: 26.45 (7.58, 1.32, 7.85, 7.95, 1.75), palp: 6.10 (2.15, 0.77, 2.44, -, 0.74). Carapace brown, with black brown radiating stripes and longitudinal bands marginally, highest at the center, covered by dense hairs posteriorly, and with no thoracic furrow (Figure 15C). Six eyes follow the generic pattern. Eye diameters: PME 0.26; ALE 0.26; PLE 0.26. Chelicerae without conspicuous obvious spaced stridulatory ridges (Figure 20B). Fangs, endites, labium, sternum, and spinnerets follow the generic pattern. Habitus as in Figure 15C. Legs yellowish, relatively long and slender, with spines on leg Ⅰ and leg Ⅱ, and claws follow the generic pattern. Opisthosoma slightly yellowish dorsally, without regular markings. Colulus trapeziform. Palps as in Figure 14A-F; palp with a long embolus, apically aciculate; tarsus smaller than or subequal to the basal portion of the bulb; apex of tarsus covered with dense cluster of hairs.

**Female:** Total length 9.41, prosoma 5.16 long, 4.05 wide, opisthosoma 4.25 long, 3.48 wide, chelicerae 1.24, labium 1.05 long, 0.68 wide, sternum 2.10 long, 1.85 wide. Leg Ⅰ: 27.63 (7.86, 1.19, 8.25, 8.75, 1.58), leg Ⅱ: -(7.15, 1.24, 7.25, 6.49, -), leg Ⅲ: 18.17 (5.28, 1.15, 4.95, 5.44, 1.35), leg Ⅳ: 23.68 (6.95, 1.25, 7.14, 6.85, 1.49), palp: 4.11 (1.15, 0.66, 1.05, -, 1.25). Prosoma chestnut brown, fovea absent, with dark brown radiating stripes and reddish bands marginally, highest at the center, and with shallow thoracic furrow (Figure 15D). Eye diameters: PME 0.26; ALE 0.26; PLE 0.26. Chelicerae with 3 conspicuous well-spaced stridulatory ridges mesally, 4 short inconspicuous ones distally (Figure 20A). Other characters similar to male except for the color, which is browner on opisthosoma and legs of the female. Habitus as in Figure 15D-E. Legs yellowish-brown, long and slender, without rings and spines. Palps, claws and spinnerets follow the generic pattern; colulus oval, conspicuous. The spermathecae interdistance is 1.43 (Figure 15B).

**Distribution.** Known only from type locality in Guangxi, China (Figure 22).

**Natural History.** The species was found in the aphotic zone, far from the entrance of the long, humid cave, hanging on its web.

***Stedocys zhaoi* Wu & Li sp. nov.**

Figures 16-17, 20C-D

**Type material. Holotype:** ♂, unnamed cave (N14°12.189′, E99°01.701′, 185 m a.s.l.), Wang Krachae Subdistrict, Sai Yok District, Kanchanaburi, Thailand, 01.10.2014, H. Zhao, Y. Li and Z. Chen. **Paratypes:** 5♀, same data as holotype.

**Etymology.** The specific name is a patronym in honor of the collector Huifeng Zhao; noun (name) in genitive case.

**Diagnosis.** The species can be easily distinguished from all known congeners by the coiled embolus distally (Figure 16A-F), the conspicuous arcuate sclerotized structure of the female external genitalia (Figure 17A), and by the two pairs of round spermathecae on long, curved stalks (Figure 17B).

**Description. Male (holotype):** Total length 9.89, carapace 4.95 long, 4.13 wide, opisthosoma 4.94 long, 3.48 wide, chelicerae 1.23, labium 1.53 long, 0.58 wide, sternum 2.48 long, 1.74 wide. Leg Ⅰ: 36.09 (10.75, 1.75, 11.27, 10.79, 1.53), leg Ⅱ: 33.25 (10.17, 1.85, 10.39, 9.48, 1.36), leg Ⅲ: 21.79 (6.85, 1.25, 6.25, 6.35, 1.09), leg Ⅳ: -(9.47, 1.35, -, -, -), palp: 4.05 (1.63, 0.55, 1.24, -, 0.63). Carapace chestnut brown with orange radiating stripes, highest at the center, covered by slender hairs posteriorly, with a shallow, orange thoracic furrow (Figure 17C). Six eyes follow the generic pattern. Eye diameters: PME 0.28; ALE 0.29; PLE 0.31. Chelicerae with 6 well-spaced stridulatory ridges mesally, and 3 shorter inconspicuous ones distally (Figure 20D) Fangs, endites, labium sternum, colulus and spinnerets follow the generic pattern. Habitus as in Figure 17C. Legs yellowish, without annulations or spines, three pretarsal claws. Opisthosoma slightly whitish-yellow dorsally, the first half with two irregular pairs of brown marks extending laterally and four pairs of chevron-like marks on posterior half. Palps as in Figure 16A-F; palp with tarsus smaller than the tegulum; the apex of the tarsus covered with a dense cluster of hairs (Figure 16A).

**Female:** Total length 11.12, carapace 4.86 long, 3.98 wide, opisthosoma 6.25 long, 5.75 wide, chelicerae 1.43, labium 1.15 long, 0.55 wide, sternum 2.05 long, 1.15 wide. Leg Ⅰ: 35.02 (10.55, 1.45, 11.05, 10.49, 1.48), leg Ⅱ: 31.79 (9.53, 1.35, 9.95, 9.47, 1.49), leg Ⅲ: -(6.77, 1.25, -, -, -), leg Ⅳ: 23.55 (8.75, 1.25, 6.25, 6.25, 1.05), palp: 4.27 (1.24, 0.57, 0.98, -, 1.48). Carapace similar to male (Figure 17D). Six eyes follow the generic pattern. Eye diameters: PME 0.28; ALE 0.29; PLE 0.29. Chelicerae with 5 well-spaced stridulatory ridges mesally, and 3 short inconspicuous ones distally (Figure 20C). Other characters are similar to male except for the markings and color whihc is lighter in the opisthosoma. Habitus as in Figure 17D-E. Legs yellowish-brown. Palps and spinnerets follow the generic pattern; colulus oval. Vulva (Figure 17B) with two pairs of spermathecae, with the innermost spermathecae 1.5 times as large as the outermost.

**Distribution.** Known only from type locality in Kanchanaburi, Thailand (Figure 22).

**Natural History.** The species was found in the aphotic zone of the cave.
